# Efficacy and safety of continuous 4-year telbivudine treatment in patients with chronic hepatitis B

**DOI:** 10.1111/jvh.12025

**Published:** 2012-12-27

**Authors:** Y Wang, S Thongsawat, E J Gane, Y-F Liaw, J Jia, J Hou, H L Y Chan, G Papatheodoridis, M Wan, J Niu, W Bao, A Trylesinski, N V Naoumov

**Affiliations:** 1 Institute of Infectious Diseases, Southwest Hospital, Third Military Medical UniversityChongqing, China; 2 Department of Internal Medicine, Faculty of Medicine, Chiang Mai UniversityChiang Mai, Thailand; 3 New Zealand Liver Unit, Auckland City HospitalAuckland, New Zealand; 4 Liver Research Unit, Chang Gung Memorial Hospital & UniversityTaipei, Taiwan; 5 Liver Research Center, Beijing Friendship Hospital, Capital Medical UniversityBeijing, China; 6 Hepatology Unit and Department of Infectious Diseases, Nanfang HospitalGuangzhou, China; 7 Medicine and Therapeutics, Chinese University of Hong KongHong Kong, China; 8 Academic Department of Medicine, Hippokration General HospitalAthens, Greece; 9 Department of Infectious Diseases, ChangHai Hospital of the Second Military Medical UniversityShanghai, China; 10 Department of Hepatology, First hospital of Jilin UniversityJilin, China; 11 Novartis Pharma CorporationEast Hanover, NJ, USA; 12 Novartis Pharma AGBasel, Switzerland

**Keywords:** HBeAg seroconversion, long-term treatment, off-treatment, renal function, safety

## Abstract

In the phase-III GLOBE/015 studies, telbivudine demonstrated superior efficacy *vs* lamivudine during 2-year treatment in HBeAg-positive and HBeAg-negative chronic hepatitis B (CHB). After completion, 847 patients had an option to continue telbivudine treatment for further 2 years. A total of 596 (70%) of telbivudine-treated patients, who were serum HBV DNA positive or negative and without genotypic resistance to telbivudine at the end of the GLOBE/015 trials, were enrolled into a further 2-year extension study. A group of 502 patients completed 4 years of continuous telbivudine treatment and were included in the telbivudine per-protocol population. Amongst 293 HBeAg-positive patients, 76.2% had undetectable serum HBV DNA and 86.0% had normal serum ALT at the end of 4 years. Notably, the cumulative rate of HBeAg seroconversion was 53.2%. Amongst 209 HBeAg-negative patients, 86.4% had undetectable HBV DNA and 89.6% had normal serum ALT. In patients who had discontinued telbivudine treatment due to HBeAg seroconversion, the HBeAg response was durable in 82% of patients (median 111 weeks of off-treatment follow-up). The cumulative 4-year resistance rate was 10.6% for HBeAg-positive and 10.0% for HBeAg-negative patients. Most adverse events were mild or moderate in severity and transient. Renal function measured by estimated glomerular filtration rate (eGFR) increased by 14.9 mL/min/1.73 m^2^ (16.6%) from baseline to 4 years (*P* < 0.0001). In conclusion, in HBeAg-positive and HBeAg-negative CHB patients without resistance after 2 years, two additional years of telbivudine treatment continued to provide effective viral suppression with a favourable safety profile. Moreover, telbivudine achieved 53% of HBeAg seroconversion in HBeAg-positive patients.

## Introduction

Of the five oral nucleos(t)ide analogues currently available for the treatment of chronic hepatitis B virus (HBV) infection, the newer agents (entecavir, telbivudine and tenofovir) induce potent suppression of HBV replication. Practice guidelines recommend the monitoring of on-treatment serum HBV DNA levels at 24 weeks of therapy with nucleos(t)ide analogues as a ‘roadmap’ strategy to manage and improve outcomes in patients with chronic hepatitis B (CHB); all PCR-positive patients on telbivudine or lamivudine beyond Week 24 should have a treatment adaptation 1–5. Amongst the other key markers of treatment response, hepatitis B e antigen (HBeAg) seroconversion is an important event in the natural course of CHB 2,4,5.

Telbivudine provides effective treatment of patients with CHB, as shown in randomized, double-blind, multicenter phase 3 trials. The GLOBE trial, the largest intent-to-treat analysis of oral antiviral treatment in patients with CHB, demonstrated the superiority of telbivudine over lamivudine in all efficacy measures 6,7. Study 015, similar in design as the GLOBE trial, was conducted in China and also confirmed the greater antiviral and clinical efficacy of telbivudine 8. However, there is limited information on treatment outcomes and predictors of response beyond 2 years of therapy for each new oral nucleos(t)ide analogue and for telbivudine in particular 9–11.

This study investigated the safety and efficacy of long-term telbivudine treatment in the group of patients without genotypic resistance after 2 years of telbivudine in the GLOBE and 015 studies during 4 years of continuous telbivudine treatment and assessed the durability of virologic responses in patients who discontinued telbivudine because they had achieved predefined efficacy parameters.

## Methods

### Study design and patients

This was an open-label study of telbivudine treatment in patients with chronic hepatitis B who had been previously treated for 2 years in the GLOBE and 015 feeder studies (extension study 2303).

At the end of the feeder studies, patients had the option to stop therapy, switch to another treatment, or enrol in this open-label extension in which all patients continued treatment with telbivudine 600 mg/day. Only patients treated with telbivudine in the feeder studies and enrolled in this extension study were considered for the 4-year telbivudine long-term analysis (with PCR-undetectable or PCR-detectable serum HBV DNA). Patients with documented genotypic viral resistance at study entry (Week 104 of the feeder study) were not eligible for extended treatment with telbivudine and were not included in the efficacy analysis (patients with genotypic viral resistance were nevertheless analysed for safety). It was planned that patients enrolled in this extension study received 2 years of additional telbivudine 600 mg/day and therefore a total of 4 years of continuous telbivudine treatment.

All 847 patients treated with telbivudine in the GLOBE and 015 studies had the opportunity to roll over into study 2303; however, 114 patients (32 HBeAg-negative and 82 HBeAg-positive) patients did not enrol – either at their own or the investigator's discretion. These patients were included in the 2-year ITT analysis of the GLOBE study; 46 were PCR-negative, 68 were PCR-positive, 59 had ALT normalization and 17 had HBeAg seroconversion, with no additional data available. Another 137 patients were enrolled but not eligible for efficacy assessment due to genotypic resistance at Week 104 of the feeder studies; however, their safety is reported in the safety population. Of the 251 patients who were not enrolled into study 2303, their baseline virologic characteristics and ALT levels were similar to those of the enrolled patients.

**Fig. 1:**
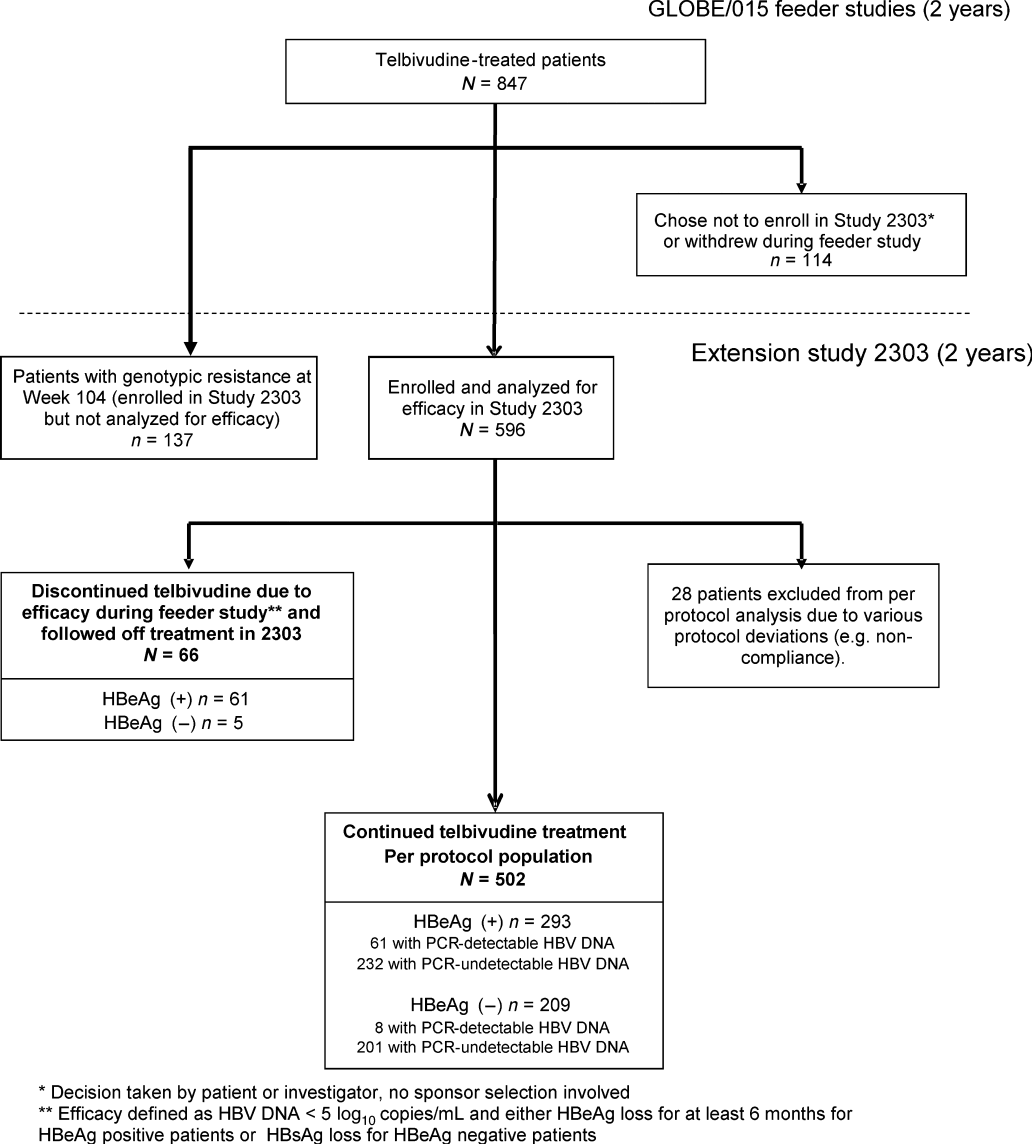
Patient flow diagram.

Altogether, 596 of 847 (70%) telbivudine-treated patients were analysed for efficacy in the extension study 2303: 66 patients enrolled into the off-treatment follow-up group and 530 (89%) patients continued telbivudine 600 mg/day treatment for 2 more years (Fig. 1). Of the 530 patients, 28 patients were excluded from per-protocol analysis due to protocol deviations (e.g. noncompliance). The per-protocol population in study 2303 consisted of 502 patients (293 HBeAg-positive and 209 HBeAg-negative). Amongst the per-protocol patients, 61 of 293 (21%) HBeAg-positive patients and eight of 209 (4%) HBeAg-negative patients had PCR-detectable serum HBV DNA at entry to study 2303 (Fig. 1). Of the 293 patients who were HBeAg-positive at entry to the feeder studies, 130 had HBeAg loss by the start of the 2303 study (i.e. after the first 2 years of telbivudine treatment).

In addition, 66 telbivudine-treated patients (61 HBeAg-positive and 5 HBeAg-negative) who participated in the GLOBE/015 studies and had discontinued treatment due to efficacy following the investigator's decision based on protocol-specified criteria before Week 104 [i.e. patients who received ≥52 weeks of therapy, had serum HBV DNA levels <5 log_10_ copies/mL (4.28 log_10_ IU/mL) and demonstrated HBeAg loss/seroconversion for ≥6 months (HBeAg-positive patients) or achieved HBsAg loss (HBeAg-negative patients) before Week 104] were enrolled in the off-treatment follow-up arm of this study (Fig. 1).

Furthermore, efficacy data of the lamivudine-treated patients who participated in the GLOBE/015 feeder studies and had switched to telbivudine in the extension study, with negative PCR at the time of switch, are also presented.

Written informed consent was obtained from each patient to participate in the above-mentioned studies. The protocols were conducted in accordance with the Declaration of Helsinki and were approved by each local independent Ethics Committee. The studies were registered with the http://ClinicalTrials.gov identifiers NCT00057265 (Study GLOBE), NCT00131742 (Study 015) and NCT00142298 (Extension Study 2303).

### Efficacy assessment

Efficacy measures included the proportion of patients with undetectable serum HBV DNA [<300 copies/mL (57 IU/mL)] by PCR, normalization of ALT levels [≤1 × the upper level of normal (ULN)], HBeAg loss, HBeAg seroconversion, hepatitis B surface antigen (HBsAg) loss, HBsAg seroconversion and genotypic resistance. HBeAg seroconversion was defined as the cumulative rate over 4 years of therapy for patients who continued telbivudine treatment.

Off-treatment efficacy analyses included HBeAg loss/seroconversion and HBV DNA levels evaluated at the end of treatment and the end of off-treatment follow-up for those patients who had discontinued due to efficacy involving HBeAg loss/seroconversion. Multivariate analysis on efficacy outcomes was performed, including baseline body mass index, ALT, HBV DNA, gender, Age, Knodell HAI, genotype (C *vs* non-C), Ishak fibrosis score, race (Causasian, Asian, others) and Week-24 HBV DNA [<300 *vs* ≥300 copies/mL (57 IU/mL)].

The group of patients, who switched from lamivudine (2-year treatment during feeder studies GLOBE/015) to telbivudine during study 2303, were evaluated for HBV DNA levels, HBeAg loss/seroconversion and viral failure [HBV DNA ≥1000 copies/mL (190 IU/mL) at a visit].

Standardized tests were performed centrally by Quintiles Transnational (Research Triangle Park, NC). Serum HBV DNA levels were quantified by COBAS® Amplicor HBV Monitor PCR assay (Roche Molecular Systems, Pleasanton, CA, USA), which has a detection limit of 300 copies/mL (conversion factor: 1 IU = 5.26 copies).

### Resistance analysis

As reported previously, resistance was defined as the emergence of treatment-associated resistance mutations, identified by direct sequencing of the HBV polymerase gene at Weeks 156 and 208 (or at last available assessment if a patient discontinued prematurely) in all patients with serum HBV DNA levels >1000 copies/mL (190 IU/mL) and compared with the HBV DNA sequences at baseline 7. DNA sequencing was performed at an independent laboratory (Delft Diagnostic Laboratory, Rijswijk, the Netherlands).

For the calculation of genotypic resistance rates, a formula has been adapted based on the recommendation by an international panel of experts 12. According to the current telbivudine label, PCR-positive patients should receive alternate treatment 1. Therefore, only PCR-negative patients at the end of the previous year are counted at each time interval. The incidence of resistance is reported as adjusted cumulative probability of occurrence using the formula of Pawlotsky *et al*.: *P* = 1 – (1 – *a*_1_/*n*_1_)(1 − *a*_2_/*n*_2_)…(1 – *a*_*i*_/*n*_*i*_), where *P* is the cumulative probability that the event will occur, a_*i*_ is the number of cases at year *i* and *n*_*i*_ is the number of patients still followed up at year *i* 12,13.

### Safety assessment

The safety population was larger and consisted of patients with or without documented genotypic viral resistance at Week 104 (*n* = 655). ALT flares were defined as ALT elevation ≥10 × ULN and ≥2 × baseline value, as per the American Association for the Study of Liver Diseases (AASLD) practice guidelines 5. Grade 3–4 creatine kinase (CK) elevations were defined as CK levels >7 × ULN. Renal function was assessed using the MDRD (Modification of Diet in Renal Disease) formula for the calculation of estimated glomerular filtration rate (eGFR) 14.

### Statistical analysis

As the 4-year analysis is intended to summarize efficacy and safety in patients continuously treated with telbivudine monotherapy, no statistical comparison between treatment groups was performed.

Analyses of 4-year efficacy results [HBV DNA <300 copies/mL (57 IU/mL)], ALT normalization, HBeAg and HBsAg loss/seroconversion) were based on all data observed and missing data were not imputed. The cumulative HBeAg seroconversion rate was defined as the percentage of HBeAg-positive patients with documented seroconversion at any point during the 4-year treatment period, including patients who subsequently seroreverted.

For multivariate analysis, stepwise regression methods were applied to select the variables in the logistic regression model (*P* ≤ 0.1 to enter, *P* ≤ 0.1 to stay).

Analysis of the off-treatment durability of efficacy responses included all patients from the GLOBE/015 studies who discontinued treatment due to efficacy and were enrolled in the off-treatment follow-up arm of this study. Last observation carried forward (LOCF) method was used for the off-treatment population to impute for missing assessments.

Analysis of patients from the lamivudine arm in the feeder study, who switched to telbivudine in the extension study and PCR-negative at the time of switch were analysed using the LOCF method.

## Results

### Virologic and biochemical responses over 4 years

Demographic and other baseline characteristics of the per-protocol population of 502 patients were shown in Table 1. Efficacy results at Week 208 of telbivudine treatment in the per-protocol population of 502 patients without genotypic resistance after 2-years of telbivudine treatment in the feeder studies were shown in Table 2. The per-protocol population of 502 patients who received continuous telbivudine treatment had high rates of viral suppression [HBV DNA <300 copies/mL, (57 IU/mL)] and serum ALT normalization after 4 years of treatment. HBeAg-negative patients had higher rates of viral suppression than HBeAg-positive patients (86.0% *vs* 76.2%, respectively). Notably, the rate of undetectable viraemia increased further (i) in patients with early viral suppression by Week 24 (87.9% of HBeAg-positive patients and 87.5% of HBeAg-negative patients), (ii) in HBeAg-positive patients with early viral suppression by Week 24 together with ALT ≥2 × ULN and HBV DNA <9 log_10_ copies/mL (8.3 log_10_ IU/mL) at feeder study baseline (93.3%) and (iii) in HBeAg-negative patients with early viral suppression by Week 24 together with HBV DNA <7 log_10_ copies/mL (6.3 log_10_ IU/mL) at feeder study baseline (88.3%).

**Table 1:** Demographic and other baseline characteristics of the per-protocol population

	HBeAg-positive *N* = 293	HBeAg-negative *N* = 209
Age (years), mean (SD)	29.0 (9.3)	41.3 (11.1)
Gender, *n* (%)		
Male	221 (75.4)	164 (78.5)
Female	72 (24.6)	45 (21.5)
Race, *n* (%)		
Caucasian	20 (6.8)	35 (16.7)
Asian	265 (90.4)	153 (73.2)
African/African American	0	3 (1.4)
Hispanic/Latino	0	1 (0.5)
Middle Eastern/Indian	3 (1.0)	5 (2.4)
Other	5 (1.7)	12 (5.7)
HBV genotype, *n* (%)		
A	6 (2.0)	7 (3.3)
B	97 (33.1)	53 (25.4)
C	168 (57.3)	102 (48.8)
D	20 (6.8)	42 (20.1)
Other/unknown	2 (0.7)	5 (2.4)
HBV DNA (log_10_ copies/mL), median (range)	9.4 (3–16)	7.1 (3–13)
ALT (IU/L), median (range)	116.0 (22–623)	98.0 (31–569)

ALT, alanine aminotransferase; HBeAg, hepatitis B e antigen; HBV, hepatitis B virus.

**Table 2:** Efficacy results at Week 208 of telbivudine treatment in the per-protocol population of 502 patients without genotypic resistance after 2-years of telbivudine treatment in the feeder studies

					HBV DNA <300 copies/mL at Week 24	
		
	All patients		HBV DNA <300 copies/mL at Week 24		Baseline HBVDNA <9 log_10_ and ALT ≥2 × ULN	Baseline HBV DNA <7 log_10_
			
Week 208 Results	HBeAg-positive *N* = 293	HBeAg-negative *N* = 209	HBeAg-positive *N* = 162	HBeAg-negative *N* = 179	HBeAg-positive *N* = 39	HBeAg-negative *N* = 94
HBV DNA level <300 copies/mL, *n* (%)	163/214 (76.2)	141/164 (86.0)	109/124 (87.9)	126/144 (87.5)	28/30 (93.3)	68/77 (88.3)
ALT normalization, *n* (%)	178/206 (86.4)	129/144 (89.6)	107/120 (89.2)	112/126 (88.9)	27/30 (90.0)	56/61 (91.8)

ALT, alanine aminotransferase; HBeAg, hepatitis B e antigen; HBV, hepatitis B virus. The denominator for each parameter reflects the number of patients available for assessment.

Multivariate analysis in HBeAg-positive patients on parameters influencing undetectable viraemia at 4 years confirmed Week-24 undetectable HBV DNA (odds ratio 3.11, *P* = 0.0023) as independent significant positive predictor, whilst baseline HBV DNA <9 log_10_ copies/mL (8.3 log_10_ IU/mL) (odds ratio 2.27) was not significant (*P* = 0.0646).

The levels of ALT normalization remained high (>86%), regardless of HBV DNA levels at Week 24 or feeder study baseline characteristics.

**Fig. 2:**
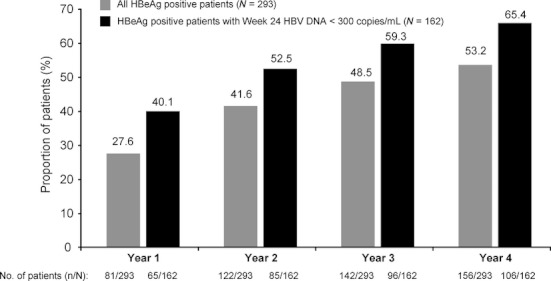
Cumulative HBeAg seroconversion rates over time in telbivudine-treated HBeAg-positive patients without genotypic resistance after Year 2 (all HBeAg-positive patients and HBeAg-positive patients who had HBV DNA <300 copies/mL at Week 24).

The cumulative rate of HBeAg seroconversion increased over time from 27.6% at Year 1 to 53.2% at Year 4 in HBeAg-positive patients on continued telbivudine treatment (Fig. 2, grey bars). Higher cumulative rates of HBeAg seroconversion were seen in patients who achieved undetectable HBV DNA levels at Week 24 (Fig. 2, black bars). Week-24 undetectable HBV DNA was found as significant independent positive predictor (odds ratio 3.17, *P* < 0.0001) for HBeAg seroconversion in multivariate analysis.

At Week 208, HBsAg loss was achieved by 4/213 (1.9%) HBeAg-positive and 1/164 (0.6%) HBeAg-negative patients. Of these patients, 1/213 (0.5%) and 0/164 were able to achieve HBsAg seroconversion at Week 208, respectively.

### Off-treatment durability of efficacy responses

At the end of the GLOBE/015 studies, 66 patients met the per-protocol criteria for discontinuation of telbivudine treatment due to efficacy (see definition in Patients and Methods) (Fig. 1). Before treatment discontinuation, 98% of patients had undetectable HBV DNA [<300 copies/mL (57 IU/mL)] and all had HBeAg seroconversion.

After a median of 111 weeks off-treatment, 50 of the 61 HBeAg-positive patients (82%) sustained HBeAg seroconversion. At their last follow-up visit, they had a median HBV DNA of 3.56 log_10_ copies/mL (2.83 log_10_ IU/mL) (14 patients had undetectable HBV DNA, 28 patients had <4 log_10_ copies/mL HBV DNA (3.27 log_10_ IU/mL), and all of which had normal ALT). Four patients in the off-treatment follow-up arm achieved HBsAg loss. Kaplan–Meier estimation showed that 81.3% of patients had a durable response during more than 2 years of off-treatment follow-up.

### Patients who switched from lamivudine to telbivudine

Of the 852 patients treated with lamivudine in the feeder Phase 3 studies, 398 without genotypic resistance at the end of the feeder studies rolled over to extension study 2303 and received telbivudine for two additional years. Of these, 299 (171 HBeAg-positive and 128 HBeAg-negative) had undetectable HBV DNA at the time of the switch and were analysed for efficacy. After 2 years of telbivudine switch, of the 171 HBeAg-positive patients, 92% (157/170) maintained undetectable HBV DNA, 89% (144/162) had ALT normalization, 76% (120/158) HBeAg loss and 54% (85/158) HBeAg seroconversion. The rate of viral failure [HBV DNA ≥1000 copies/mL (190 IU/mL)] was 5% (8/170). Of the 128 HBeAg-negative patients, 92% (117/127) maintained undetectable HBV DNA and 85% (99/117) had ALT normalization. The rate of viral failure was 6% (7/127).

### Viral breakthrough and genotypic resistance

In the overall patient population, treated continuously with telbivudine during studies GLOBE/015 and the 2-year extension study (*n* = 502), only patients without genotypic resistance at the end of the feeder studies continued treatment with telbivudine in Years 3 and 4. When the method recommended by the international expert panel was applied to per-protocol population without viral resistance during the first 2 years, the cumulative viral breakthrough/resistance rates in HBeAg-positive patients (corresponding to 13 and 11 cases of genotypic resistance) were 10.6%/5.6% and 18.8%/10.6% during Years 3 and 4, respectively.^1^ Similarly, in HBeAg-negative patients (based on 10 and 9 patients with genotypic resistance), the cumulative viral breakthrough/resistance rates were 8.4%/5.0% and 15.9%/10.0% during Years 3 and 4, respectively.^2^

In telbivudine-treated HBeAg-positive patients with baseline HBV DNA <9 log_10_ copies/mL (8.3 log_10_ IU/mL) and ALT ≥2 × ULN and HBeAg-negative patients with baseline HBV DNA <7 log_10_ copies/mL (6.3 log_10_ IU/mL), together with undetectable HBV DNA at Week 24 from the feeder studies, resistance was assessed applying the mathematical model to this defined population followed in Years 3 and 4. In this subgroup, for HBeAg-positive patients, 2 of 37 and 2 of 32 patients developed genotypic resistance during Years 3 and 4, respectively. The cumulative numbers of patients with genotypic resistance in HBeAg-negative patients were 7 of 91 and 3 of 77 patients during Years 3 and 4, respectively.

### Safety and tolerability

The safety population consisted of 655 patients (with or without genotypic resistance at Week 104). The safety and tolerability profile of telbivudine over 4 years was similar to that reported at 3 years in patients from the GLOBE study 11. Most adverse events were mild or moderate in severity and transient, and no new safety signals were observed. Over the 4 years, adverse events were reported by the investigator to be possibly related to study drug in 194 (29.6%) patients in the safety population.

The most common drug-related adverse events consisted of increased serum creatine kinase (CK) (10.1%), headache (2.9%), nausea (2.0%) and fatigue (2.7%). Infection-related adverse events were reported in 34 patients (5.2%) and consisted primarily of hepatitis B worsening or flare (14 patients; 2.1%).

Muscle symptoms were reported in 40 (6.1%) patients (Table 3). For one patient with ‘myopathy’, the EMG reported no myopathic features and he completely recovered. The other patient reported as ‘myopathy’ improved on treatment by the last follow-up time in the study. Two patients were reported with myositis (grade 1) and classified as unrelated with telbivudine by investigators, and telbivudine was not discontinued. One completely recovered, the other improved by the last available follow-up time.

**Table 3:** Incidence of muscle symptoms and neuropathy events over 4 years in the telbivudine-treated safety population (*n* = 655)

	*n* (%) of patients*
Any adverse event	548 (83.7) *
Muscle symptoms, total	40 (6.1)
Myalgia	30 (4.6)
Muscular weakness	6 (0.9)
Musculoskeletal pain	2 (0.3)
Myopathy	2 (0.3)
Myositis	2 (0.3)
Musculoskeletal discomfort	1 (0.2)
Peripheral neuropathy, total	8 (1.2)
Paraesthesia	4 (0.6)
Neuralgia	1 (0.2)
Peripheral neuropathy	1 (0.2)
Polyneuropathy	1 (0.2)
Sensory loss	1 (0.2)

* Cumulative number of patients with events over 4 years. The same patient could experience multiple events, but each patient is counted only once for the same event.

Peripheral neuropathy was reported in 8 (1.2%) patients, with half of these patients experiencing paraesthesia (Table 3). Five of the eight patients completely recovered during the study without stopping telbivudine; three patients improved by the last available follow-up time in the study.

Drug discontinuation related to an adverse event was reported for 18 patients (2.7%): investigation-related adverse events (six patients) and infections (four patients) consisting primarily of hepatitis B flare or exacerbation were the most common. In three patients (one with myopathy, one with polymyositis and one with spinal muscular atrophy), muscle adverse events led to discontinuation.

Over the course of treatment, median CK increased from 101 IU/mL (baseline of feeder studies) to 172 IU/mL (4 years). Grade 3-4 CK elevations were reported for 104 (15.9%) patients, but most of them were transient (97.5% lasting one or two visits) and spontaneously recovered (93.2% resolved or returned to baseline levels during the study period).

Cumulatively, over 4 years, 42 (6.4%) patients had an ALT flare (as defined previously by 2007 AASLD guideline) 5. In 29 of the 42 patients, the ALT flare was due to viral breakthrough. In the remaining 13 patients, ALT flare without viral breakthrough occurred early after starting treatment, around treatment Weeks 4–12, and 9 of 11 HBeAg-positive patients achieved HBeAg seroconversion.

**Fig. 3:**
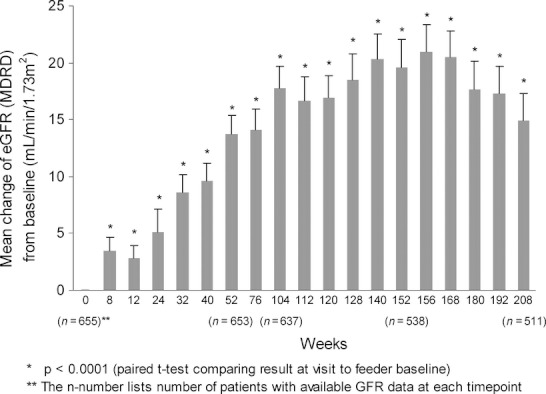
Mean changes (95% confidence interval) of estimated glomerular filtration rate (MDRD equation) from baseline during the 4-year follow-up in safety population.

Amongst telbivudine-treated patients, the eGFR as a measure of renal function increased steadily over the 2 years of the feeder study and remained at the higher level thereafter. The mean eGFR changes from baseline (94.9) to Weeks 104 (112.3) and 208 (109.9) were a significant improvement of 17.8 (*P* < 0.0001) and 14.9 (*P* < 0.0001) (MDRD formula in mL/min/1.73 m^2^), respectively (Fig. 3). This refers to 16.6% of eGFR change from baseline over 4 years. In the subpopulation of telbivudine-treated patients with mildly reduced baseline eGFR of 60-90 mL/min/1.73 m^2^, 74% (165/223) of them shifted to >90 mL/min/1.73 m^2^ after 4 years of treatment.

In patients treated with lamivudine in the feeder studies and switched to telbivudine in study 2303, the general safety profile was very similar to the population described previously and is not described in detail. During 2 years of lamivudine treatment, eGFR increased slightly from 93.95 to 99.50 mL/min/1.73 m^2^ (8.9%), whilst an additional increase in 11.93 mL/min/1.73 m^2^ (9.6% *vs* time of switch) was noted after the switch to telbivudine for two additional years.

## Discussion

This study with long-term telbivudine treatment in adult patients with CHB demonstrates the safety and efficacy of telbivudine 600 mg during 4-year treatment of HBeAg-positive and HBeAg-negative patients. The current cohort analysis includes the majority of telbivudine-treated patients from the Phase 3 studies GLOBE/015; 596 of all 847 telbivudine-treated patients (70%) were enrolled in the extension study 2303.

Continuous telbivudine treatment over 4 years resulted in high rates of undetectable HBV DNA (76.2% for HBeAg-positive and 86.0% for HBeAg-negative patients) and ALT normalization (86.4% and 89.6%, respectively). Previous studies have shown that maximal suppression of HBV replication improves efficacy outcomes as measured by virologic and serologic markers. We have previously identified that undetectable HBV DNA at Week 24 is the most significant predictor of response after 2 years treatment with telbivudine 15. Thus, the highest rates of HBeAg seroconversion and lowest rates of antiviral drug resistance have been observed in patients who were able to achieve serum HBV DNA levels below the limit of detection in the first 24 weeks of antiviral therapy 6,7,16–18. In our follow-up cohort, we confirmed Week-24 undetectable HBV DNA as a significant predictor of long-term outcome in HBeAg-positive patients achieving higher rates of viral suppression and HBeAg seroconversion. The value of the Week-24 decision point has been recently confirmed prospectively in ‘telbivudine roadmap studies’ achieving 94% undetectable HBV DNA and 44% HBeAg seroconversion using tenofovir add-on therapy only for patients with detectable HBV DNA at Week 24 19,20.

The population reported in this manuscript is a selected population and includes patients who received telbivudine continuously for 4 years, excluding patients who developed genotypic resistance after the initial 2 years. This is in line with current treatment guidelines as well as the telbivudine label, recommending treatment modifications for patients with genotypic resistance on telbivudine 1,2,5. Treatment intensification was not provided at any time during the GLOBE or 015 studies. Similarly, there was no intensification in the 2303 extension study (as this was designed prior to 2009 practice guidelines). The risk of developing resistance to oral nucleos(t)ide treatment increases with time if serum HBV DNA remains positive, whilst the risk of resistance is markedly reduced in patients with an early viral response and undetectable HBV DNA at treatment Week 24. This led to the ‘roadmap concept’ during telbivudine treatment with monitoring of HBV DNA levels at 24 weeks and every 6 months thereafter with the addition of a nucleos(t)ide analogue without cross resistance (such as adefovir dipivoxil or tenofovir) if viremia is present to reduce the risk of resistance 21. In the present study, we used the method recommended by an international panel of experts to calculate cumulative resistance rates for telbivudine in patients from the GLOBE/015 trials enrolled in the 2303 extension study, as this method was previously applied to calculate the resistance to entecavir, adefovir and other antivirals 12,13,22. For patients who have positive HBV DNA at Week 24 during telbivudine treatment, intensification with tenofovir or adefovir is recommended and recent prospective studies demonstrated that using the roadmap concept results in high rates of HBV DNA suppression and the prevention of resistance along with high rates of HBeAg seroconversion 19,20.

Current practice guidelines recommend discontinuation of antiviral treatment, after a consolidation period, in HBeAg-positive patients who achieve HBeAg seroconversion 2,4,5. In these patients, HBeAg seroconversion is generally associated with sustained HBV DNA suppression, ALT normalization and remission of the liver 23. In our long-term cohort with 4 years of telbivudine treatment, we observed a steady increase in cumulative rate of HBeAg seroconversion over time from 27.6% at Year 1 to 53.2% at Year 4 in HBeAg-positive patients on continued telbivudine treatment.

The durability of HBeAg seroconversion after the end of treatment with nucleos(t)ide analogues is an important issue in the management of patients with CHB as it indicates the potential for a finite treatment period in this group of patients. In the separate analysis of off-treatment durability, which involved 61 telbivudine-treated patients with HBeAg-positive CHB from the GLOBE/015 trials who discontinued treatment after HBeAg seroconversion, 81% had sustained seroconversion after a median of 111 weeks off-treatment.

HBeAg seroconversion is also a critical prerequisite for the clearance of HBsAg, which is considered the ideal outcome of treatment in CHB. At Year 4, HBsAg loss was achieved in 4/213 patients (1.9%), HBeAg-positive patients on-treatment, and for four patients in the off-treatment follow-up arm. Patients with genotype A or D are more likely to lose HBsAg compared with genotype B or C and genotypes B/C were more frequent than genotype A/D in this cohort (90% *vs* 9%, respectively). HBsAg loss rates reported in recent studies with different oral nucleos(t)ides are 8 to 26% in genotypes A or D and only ∼3% in genotype B or C 24–26.

Overall, the safety results confirm that telbivudine is safe and well tolerated in patients with compensated CHB after 4 years of continuous treatment or in patients followed off-treatment for 2 years after treatment discontinuation due to achievement of efficacy. Myopathy/myositis, which is an identified risk of telbivudine treatment, was reported in only four patients over 4 years of continuous treatment. Myalgia was the most frequent muscular adverse event (4.6%); however, muscular weakness, which is a better predictor of myopathy, was reported in only 0.9% of patients. Elevated serum CK (grade 3 or 4) was reported in 15.9% of patients over 4 years, but remained transient and improved without study drug discontinuation. An analysis performed with the same safety database of 655 patients with CHB (with or without virologic resistance at Week 104) showed that there was no correlation between on-treatment muscle events and abnormal CK 27.

A novel finding in the present study which is of significant clinical importance is that telbivudine-treated patients show a steady increase in eGFR over the 2-year feeder study, remaining at an elevated level thereafter, especially in patients with mild abnormal eGFR at baseline 2. Renal dysfunction is common in patients with advanced liver diseases, especially in decompensated HBV cirrhosis and is associated with high mortality 28. In Europe, 20% of the patients with CHB have reduced baseline eGFR of 60 to 89 mL/min/1.73 m^2^ 29. The benefit of telbivudine on eGFR is unique amongst the other nucleos(t)ides 30,31. This long-term improvement of eGFR was not related to viral suppression (in compensated patients) as confirmed by patients with viral suppression under lamivudine treatment who experienced additional eGFR increases only after switch to telbivudine. The improvement of glomerular filtration is particularly important in patients with high renal risk such as mildly impaired renal function, decompensated cirrhosis, proteinuria, glomerulonephritis, uncontrolled diabetes or concomitant nephrotoxic drugs. The mechanism of this possible renal protective effect of telbivudine remains to be investigated.

In conclusion, in HBeAg-positive and HBeAg-negative patients with CHB who do not have genotypic resistance after a 2-year treatment, two additional years of telbivudine treatment continue to maintain effective viral suppression, ALT normalization and in HBeAg-positive patients achieved 53% seroconversion. In addition, 4 years of telbivudine treatment resulted in a steady improvement in renal function measured by eGFR, and overall was associated with a favourable safety profile.

## Notes

1 As defined, 233 (Year 3) and 206 (Year 4) HBeAg-positive patients were eligible for evaluation.

2 As defined, 202 (Year 3) and 170 (Year 4) HBeAg-negative patients were eligible for evaluation.

## Abbreviations

ALT: Alanine aminotransferase
CHB: Chronic hepatitis B
CK: Creatine kinase
eGFR: Estimated glomerular filtration rate
HBeAg: Hepatitis B e antigen
HBsAg: Hepatitis B surface antigen
HBV: Hepatitis B virus
LOCF: Last observation carried forward
MDRD: Modification of Diet in Renal Disease
PCR: Polymerase chain reaction
